# Feasibility and Acceptability of a Strategy Deploying Multiple First-Line Artemisinin-Based Combination Therapies for Uncomplicated Malaria in the Health District of Kaya, Burkina Faso

**DOI:** 10.3390/tropicalmed8040195

**Published:** 2023-03-28

**Authors:** Jean Moïse Tanga Kaboré, Mohamadou Siribié, Denise Hien, Issiaka Soulama, Nouhoun Barry, Adama Baguiya, Alfred B. Tiono, Christian Burri, André-Marie Tchouatieu, Sodiomon B. Sirima

**Affiliations:** 1 Groupe de Recherche Action en Santé (GRAS), Ouagadougou 10248, Burkina Faso; m.kabore@gras.bf (J.M.T.K.);; 2 Swiss Tropical and Public Health Institute, Kreuzstrasse 2, 4123 Allschwil, Switzerland; 3 University of Basel, Petersplatz 1, 4001 Basel, Switzerland; 4 Institut de Recherche en Sciences de la Santé (IRSS), Ouagadougou 7192, Burkina Faso; 5 Medicines for Malaria Venture (MMV), 1215 Geneva, Switzerland

**Keywords:** malaria, feasibility, acceptability, multiples first-line therapies, Burkina Faso

## Abstract

(1) Background: Effective malaria case management relies on World Health Organization (WHO) recommended artemisinin-based combination therapies (ACTs), but partial resistance to artemisinin has emerged and is spreading, threatening malaria control and elimination efforts. The strategy of deploying multiple first-line therapies (MFT) may help mitigate this threat and extend the therapeutic life of current ACTs. (2) Methods: A district-wide pilot quasi-experimental study was conducted, deploying three different ACTs at the public health facility (PHF) level for uncomplicated malaria treatment from December 2019 to December 2020 in the health district (HD) of Kaya, Burkina Faso. Mixed methods, including household and health facility-based quantitative and qualitative surveys, were used to evaluate the pilot programme. (3) Results: A total of 2008 suspected malaria patients were surveyed at PHFs, of which 79.1% were tested by rapid diagnostic test (RDT) with 65.5% positivity rate. In total, 86.1% of the confirmed cases received the appropriate ACT according to the MFT strategy. The adherence level did not differ by study segment (*p* = 0.19). Overall, the compliance level of health workers (HWs) with MFT strategy was 72.7% (95% CI: 69.7–75.5). The odds of using PHF as the first source of care increased after the intervention (aOR = 1.6; 95% CI, 1.3–1.9), and the reported adherence to the 3-day treatment regimen was 82.1%; (95% CI: 79.6–84.3). Qualitative results showed a high acceptance of the MFT strategy with positive opinions from all stakeholders. (4) Conclusions: Implementing an MFT strategy is operationally feasible and acceptable by stakeholders in the health systems in Burkina Faso. This study provides evidence to support the simultaneous use of multiple first-line artemisinin combination therapies in malaria-endemic countries such as Burkina Faso.

## 1. Introduction

Despite recent progress, sub-Saharan Africa still bears the major burden of malaria [1]. In 2020, malaria was the leading cause of morbidity (39.8%), and mortality (27.4%) in Burkina Faso [2] and the country was among the six countries with the highest number of malaria cases worldwide, contributing 3.4% of all cases [1]. 

Historically, the emergence and global spread of chloroquine resistance has had a dramatic public health impact [3]. ACTs were then developed and adopted as first-line drugs for malaria treatment [4]. Typically, countries recommend a single first-line ACT for the treatment of uncomplicated malaria [5]. In Burkina Faso, two WHO-recommended ACTs, namely artesunate-amodiaquine (ASAQ) and artemether-lumefantrine (AL), were introduced in 2005 into the National Malaria Control Programme (NMCP) guidelines and have since been alternately used as first-line and second-line treatments for uncomplicated malaria treatment in PHFs. In 2017, dihydroartemisinin-piperaquine (DHA–PQP) was added to the NMCP guidelines [6], followed by pyronaridine-artesunate (PYR-AS) in 2021 [7]. From all these drugs, only AL was available at PHFs. Several studies conducted in sub-Saharan Africa demonstrated the safety and efficacy of these four ACTs [8,9,10].

Globally, the deployment of ACTs contributed substantially to reducing malaria burden [1]. However, partial resistance to artemisinin derivatives or ACT partner drugs (lumefantrine, piperaquine or amodiaquine) has recently emerged and is threatening all gains made in the past decades. It is characterized by delays in parasite clearance as first reported in Cambodia [11,12,13], and followed by increased treatment failure rates, spreading rapidly in Southeast Asia [14,15,16,17,18]. In Africa, artemisinin partial resistance associated with the presence of Pfkelch13 mutations was detected in Rwanda [19], and local independent emergence of clinically artemisinin-resistant Plasmodium falciparum was confirmed in Uganda [20]. In Burkina Faso, a recent study reported inadequate PCR-corrected efficacy (below WHO threshold) of AL at day 28 (<80%) and DHA-PQP at day 42 (<90%) [21], However, no evidence of partial resistance was confirmed [22]. 

**Figure 1:**
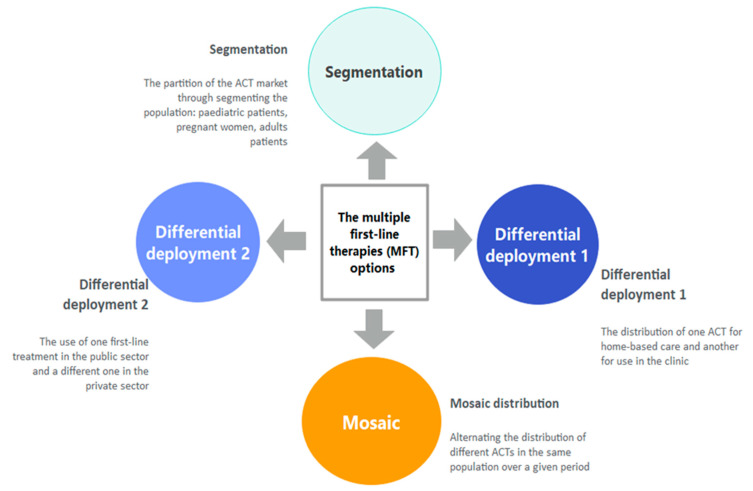
Possible options for deploying the MFT strategy.

Should ACTs lose efficacy, this would result in a dramatic rebound in malaria morbidity and mortality as no replacement drug is available. To prevent this; optimized use of the current ACTs may provide a higher long-term barrier to the emergence and/or the spread of resistance, while new drug classes are being developed. Modelling studies have concluded that deploying multiple first-line therapies (MFT) for uncomplicated malaria is a promising strategy to extend the useful therapeutic life of the current ACTs by reducing drug pressure and slowing down the spread of resistance without putting lives at risk [23,24,25,26]. The MFT strategy consists of the simultaneous use of two or more therapies combining drugs with different or opposing selection pressures for malaria case management [23]. Several options to implement MFT have been described [25,27] and summarized in Figure 1. Embedding the MFT strategy into health systems presents several challenges, including planning for the proper distribution channel of the ACTs, logistics management, and stakeholder acceptability [25]. 

To assess the feasibility and the acceptability of the MFT strategy for uncomplicated malaria case management in a high burden malaria endemic country, we conducted a pilot study in the HD of Kaya in Burkina Faso. The pilot MFT strategy consisted of deploying three different ACTs targeting three different segments of the population. Our hypothesis was that the MFT strategy could be managed by the HWs and accepted by stakeholders in the health system in Burkina Faso. This study reports on the feasibility and acceptability of this MFT pilot implementation.

## 2. Materials and Methods

### 2.1. Study Setting

**Figure 2:**
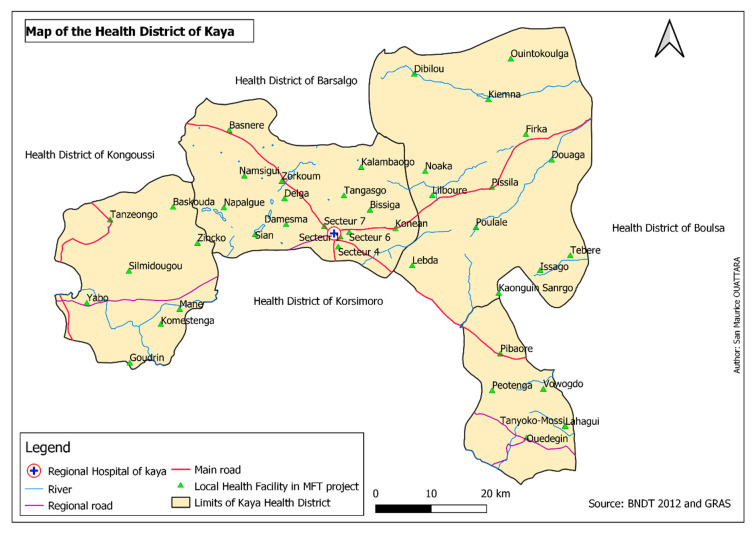
Map of health facilities in the district of Kaya included in this pilot study, 2020.

This was a district-wide pilot implementation study conducted in the health district (HD) of Kaya, in the north-central region of Burkina Faso, about 100 km from the capital city Ouagadougou. The map of the HD is presented in Figure 2. In brief, the HD comprises 43 first level PHFs, including 42 primary health care centres, one medical centre. In addition, there are four private and faith-based health facilities in Kaya. Outpatient, inpatient, vaccination, child healthcare and antenatal care services are available 24 h a day, 7 days a week. All health facilities (HF) have medicine-dispensing facilities, distributing mainly generic drugs. A free healthcare policy has been in place in Burkina Faso since April 2016 for children under 5 and pregnant women. There is in total 215 villages in an area of 3617 km^2^ with an estimated population of about 432,600 inhabitants in 2020.

### 2.2. Study Design

This was a quasi-experimental study design with pre- and post-intervention assessments conducted before the deployment of MFT and one year after the intervention, respectively. A mixed method, including desk reviews, quantitative and qualitative surveys were conducted to assess the study outcomes.

### 2.3. Description of the Intervention

The intervention took place from December 2019 to November 2020. It consisted of the deployment of three ACTs targeting three different segments of populations at the PHF level for the management of uncomplicated malaria cases. PYR–AS (Pyramax) granule formulation (20/60 mg) procured from Shin Poong (Shin Poong Pharm. Co., Ltd., Seoul, Korea) was allocated to the Segment I (children under 5 years of age); DHA–PQP (D-Artepp) tablets (40/340 mg) obtained from Fosun Pharma was allocated to Segment II (patients aged 5 years and above); and AL tablets (10/120 mg) procured from the Ministry of Health in Burkina Faso was allocated to the Segment III (pregnant women after the first trimester). All ACTs (except AL) were procured by Medicines for Malaria Venture (MMV) and prepacked in weight- and age-specific doses (Supplementary Table S1). They were administered as per manufacturer instructions, once daily for 3 days for PYR-AS and DHA-PQP, while AL was given twice a day for 3 days. 

The procurement process was aligned with the existing national system. ACTs were purchased through the central essential and generic drugs store and made available at the district drug stores. HF drug store managers supplied the required ACTs from the district store. 

The HWs were not paid for the programme implementation. The dispensation of ACTs to patients in the study area followed the routine practices. Thus, PYR-AS and AL were given free of charge to malaria patients in segments I and III, respectively. For Segment II patients, DHA-PQP were sold at a subsidised price (100 XOF (0.169 USD for six tablets)) to match the cost of generic standard treatment. 

### 2.4. Training Cascade of HWs

Before the intervention, HD and regional management teams were trained for 3 days to become trainers and supervisors. They organized thereafter 10 training sessions over 10 days for 20–30 HWs and drug store managers each. Overall, 271 HWs and all generic drug store managers (GDSM) (50) were trained on the MFT strategy, and all study-related procedures. HWs joining the HF after the initial training sessions were peer-trained onsite and during the supervision visits. 

### 2.5. Supervision

Compliance to the strategy was optimized through regular supervisions based on established procedures. Identified issues were discussed on the spot and appropriate corrective and preventative actions taken. 

### 2.6. Sampling Strategy 

#### 2.6.1. Health-Facility Based Survey

Study participants were selected through multi-stage sampling approach. First, 10 out of 33 accessible HFs were randomly selected; then, in each HF, 16 weeks of consultations out of 52 weeks were randomly selected; and lastly, all suspected malaria cases in the selected weeks were numbered and sampled using a systematic sampling method. The number of patients selected per segment and per facility was proportional to the facility’s outpatient attendance in the district. Thus, 47%, 49% and 4% of suspect malaria cases were needed for children under 5, patients aged 5 years and above, and pregnant women, respectively [28]. We included in the final sample size patients who attended the HF for fever or a history of fever. 

#### 2.6.2. Household and Qualitative Surveys

The sampling strategy for the household survey and qualitative surveys is described elsewhere [29]. Briefly, a two-stage sampling process was used for the household survey. First, a sample of the targeted village was randomly selected from the list of all the villages. Within selected villages, households were randomly visited using a “random walk” method. All individuals (children under five years of age, pregnant women, and individuals aged 5 to 15 years and individuals aged 16 to 40 years) in a household having had a fever during the past 4 weeks were interviewed directly or interviews were performed with their parents/caregivers until the sample size of the village is reached. 

Qualitative survey included in-depth individual interviews (IDI) and focus group discussions (FGDs) conducted with diverse respondents including stakeholders of the heath system and community members.

### 2.7. Sample Size

Assuming that the proportion of febrile patients tested and treated according to the MFT strategy was unknown in the framework of this new strategy and considering a design effect of 1.5; a minimum sample size of 578 patients was required to estimate the proportion of confirmed malaria patients appropriately managed with a 95% CI around the estimated proportion of 50%. This sample size was adjusted by the unknown compliance level of HWs with MFT strategy and the RDT testing and positivity rate in the district. Adjusting for 10% (representing non evaluable data from registers), the final sample size was 2001 suspected malaria patients to be sampled from the outpatient consultation registers. 

The sample size for the household survey and qualitative survey is described elsewhere [29]. Briefly, for the household surveys, the sample size was estimated at 450 participants with a confidence level of 95% and a desired power of 80%, from each of the three groups of population (children under 5, 5–15 years and 16–45 years) having had a fever. Pregnant women who had a fever were systematically included during the survey. 

The qualitative survey sampling was exhaustive for all stakeholders in the health system (at central, intermediate and peripheral level), health system managers, healthcare providers as well as community members (community leaders, adult men and heads of households, pregnant women and mothers of children under 5). 

### 2.8. Data Collection Procedures

Quantitative and qualitative data were collected using data extraction forms, household survey questionnaire and FGD, and IDI guides.

During the HF-based survey, data were extracted from the outpatient consultation registers, which captured patient characteristics, symptoms, malaria diagnosis and treatment given by trained nurses using KoboCollect on electronic tablets in February 2021. Data on study drugs and RDT supplies availability were collected monthly from all covered PHFs including information related to their availability at the start of the month, quantity received during the month, quantity dispensed to patients and expired, stock remaining at the end of the month and number of days of stock out during the month. 

Pre- and post-intervention household surveys were conducted in November 2018 and November 2020, respectively, and focused on the health-seeking behaviour of patients with fever in the preceding 4 weeks. Variables of interest included timeliness of treatment, providers of care, compliance with treatment course, and perceptions of drug effects including adverse events. 

The FGD and IDI structured interview explored the stakeholders’ perceptions and opinions about the intervention, implementation challenges, health-seeking behaviours for fever, and treatment practices. The interviews were conducted in the local language or in the language best spoken by the participant and by trained social scientists.

### 2.9. Outcomes and Explanatory Variables

The primary feasibility outcomes included adherence of HWs to the MFT guidelines [29], and study drug stock-outs. The HWs adherence was a composite indicator combining the appropriateness of ACT given (proportion of positive malaria patients treated as defined by segment according to the MFT strategy) and the correctness of the dosing regimen defined as an appropriateness of ACT given with the correct dose. The drug stock out indicator is defined as the proportion of HF stocked out for any ACT reported per month. 

The communities’ acceptability outcomes were measured during the household surveys through their adherence to the 3-day treatment regimen, the changes before and after the intervention in (i) prompt care-seeking behaviour and (ii) care-seeking at PHFs. The adherence to the treatment regimen was defined as correctly adhering if the prescribed ACT was taken for 3 days as per guidelines. Prompt care-seek behaviour was defined as seeking care within 24 h of fever onset while care-seeking at PHFs consisted of the use of PHF as the first source of providers for fever within 4 weeks before the survey. Exploratory variables included time before and after intervention variable, socio-demographic factors (age, gender, occupation, education status, and household size), and advice given to the patient regarding the dosing regimen. 

### 2.10. Analysis

Quantitative data were analysed using Stata SE V.16.1. Proportions were used for most analyses, and groups were compared using Pearson’s chi square test at significance *p*  <  0.05. Logistic regression with odds ratio and 95% confidence interval were used to measure the effect of the intervention on the promptness of care-seeking, the use of PHFs as first source of care and the community adherence to the ACT dosing regimen. Exploratory variables were selected based on rational grounds prior to analysis and included in the final model irrespective of their level of significance. Observations with missing values were excluded from the regression analysis. 

For qualitative data analysis, all interviews were transcribed in full and transcripts were checked, coded, and processed using QDA Miner Lite V2.0.8 software. Data-derived codes developed through deductive and inductive coding and retrieving were used during analysis. Thematic analysis was performed, including the acceptability of the strategy by different stakeholders, and perceptions of the limits or constraints of the strategy. 

## 3. Results

### 3.1. Quantitative Results

#### 3.1.1. HWs Compliance with MFT Strategy Guidelines

In total, 2008 suspected malaria patients were surveyed at the HF level, of which 45.1%, 50.3%, and 4.6% were in segments I, II and III, respectively. Most patients (54%) were female and lived in rural areas (59.8%) summarizes in Table 1. 

Table 2 summarizes the HW’s performance in managing malaria cases according to the study guidelines and community adherence to treatment regimen. There was 79.1% (1588/2008) of suspected malaria cases that were tested using RDT with 65.5% positivity rate. Nearly all confirmed cases (96.4%) were treated with an ACT. Few patients (2.1%) received malaria treatment despite negative RDT, and for 36%, no RDT was performed. In total, 86.1% of the confirmed cases received the appropriate ACT according to the MFT strategy. The adherence level did not differ by study segment (*p* = 0.19). Overall, 72.7% (95% CI: 69.7–75.5) of cases received the correct ACT at the correct dose. 

From the household survey, overall adherence to 3-day regimen was 82.1%. Patients treated with PYR-AS (aOR = 3.1, 95% CI 1.6–6.3) or DHA-PQP (aOR = 2.0, 95% CI 1.3–3.0) were more likely to adhere to the 3-day treatment regimen compared to AL. 

#### 3.1.2. Study Drug Stock Management

RDT stock outs were reported in 2.5% (1/40) of HFs in January 2020 for 3 days and seven HFs (7/40) in October and November 2020. 

**Figure 3:**
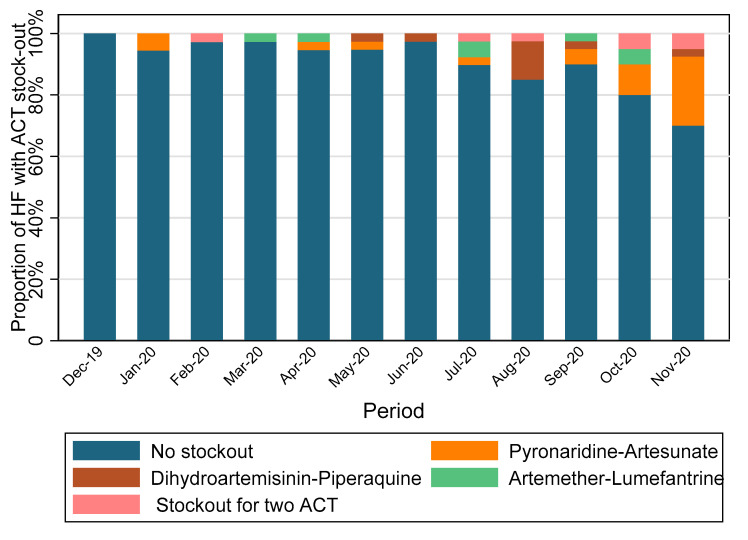
Study drugs stock management.

Figure 3 shows the study drug management during the study implementation. Overall, 9.35% of HFs stocked out for any ACT over these 12 months. None of the HFs stocked out for all three ACTs at the same time. Drug stock-outs were mostly observed during the last 4 months of study period. A total of 30% (12/40), 20% (8/40), and 10% (4/40) of HFs stocked out for any ACT in November, October, and September 2020, respectively. PYR-AS was most often stocked out. Overall, 5.70% of HFs had a stock out for PYR-AS during the study period. 

#### 3.1.3. Pre- and Post-Intervention Household Surveys

A total of 1394 and 1448 febrile patients/caregivers were interviewed at pre- and post-intervention surveys, respectively. Table 3 provides their sociodemographic characteristics. There was no difference in participants’ gender, age groups or household size between the two surveys. 

Table 4 outlines the effects of the intervention on health care seeking behaviour. The use of PHF as the first source of care significantly increased from 72.0% (95% CI; 69.6–74.4) at baseline to 79.4% (95% CI; 77.2–81.5) at the end of the study with aOR = 1.6 (95% CI, 1.3–1.9). The care-seeking behaviour for treatment in case of fever was not affected by the intervention (*p* = 0.58) conversely to the promptness which significantly decreased from 66.5% at baseline to 54.3% (*p* < 0.001). 

#### 3.1.4. Treatment Side Effects 

Throughout the intervention no serious adverse events were reported. All adverse events (AE) reported during the household survey were mild or moderate. Overall, 10.4% (99/949) patients interviewed reported having had an AE following the drug intake. The three most frequent AEs were vomiting, pruritus, and anorexia (Table 5).

### 3.2. Qualitative Results

In total, 28 IDIs and 33 FGDs were conducted post intervention. Supplementary Table S2 provides the sociodemographic characteristics of the participants interviewed.

#### 3.2.1. Health Workers’ and Policymakers’ Opinions on MFT Intervention 

HWs interviews showed that the intervention was well received. They had some good knowledge of the MFT strategy including drugs assignment to segments, dosing instructions; and found the approach straightforward to apply after appropriate training.

*“The MFT approach, I think it is good because we have three choices and what is interesting for children under 5, Pyramax is in a sachet [powder], it is in a single dose per day, and easily children accept.”*

*(IDI, Physician)*

*“For us [HWs], it does not tire us. Because once we have specified, we know that this drug is for this age group and such a treatment for such an age group. Now, you give it according to weight, quantity by weight. And since we were given the posters and put them up in the consultation rooms, I do not think there is a problem for me anyway.”*

*(FGD, head of HF)*

*“Everyone actually adheres to this new malaria treatment protocol. If you have noticed even since this morning, it is Pyramax, D-artepp except in the case of pregnant women for whom AL is given.” *

*(IDI, health worker)*

Some HWs reported that this strategy has contributed to the improvement in the care to malaria patients.

*“This strategy has contributed to better management of uncomplicated malaria. We cannot really say the opposite.”*

*(IDI, health worker)*

The policymakers also positively appreciated this new strategy.

*“This is a good strategy. I think it is new in our country. It is an experience. Thank God! Burkina Faso has given itself the power to absorb this new strategy. I followed the topic from the beginning to the end, from the training until we also report it. I think it is a good strategy”*

*(IDI, Policymaker, NMCP)*

*“It is a good approach; people have bought in.”*

*(IDI, Health authority, Head of Kaya HD)*

#### 3.2.2. Community Acceptability of the Intervention 

Community members were asked to give their perceptions of the MFT intervention. The results showed a good acceptance by the population in the study area. Most of the participants reported that they were happy with the MFT intervention. Their perceptions were based on experiences with the study drugs, including the perceived efficacy of the two new ACTs (PYR-AS and DHA-PQP).

*“These are good medicines [study drugs]; we appreciate the medicines we are given. They are effective.” *

*(FGD, Mothers of children under 5)*

*“In any case, the product [new product] treats well. In the meantime, my child had malaria, and I went to the hospital, they gave me the prescription, and I took the products, and when I gave it to the child, he quickly recovered his health.”*

*(FGD, adult men)*

#### 3.2.3. Challenges in Implementing MFT Strategy 

Despite stakeholders’ high acceptance of the intervention, some challenges and difficulties were identified, including supplies management (drug stock-outs or expiry), health staff availability, lack of refresher training for HWs, non-adherence to study guidelines.

*“In the meantime, there was stock out for D-artepp, so we had to switch to AL.”*

*(IDI, Physician)*

*“Unfortunately, several shortcomings were noted because some [local drug store managers] did not know what target to give. They did not know what was the target that was concerned in this study, what are the different prescription modalities.”*

*(IDI, Policymaker, NMCP)*

*“I went to the hospital last time and the drug was stocked out. They gave me a prescription and told me to go to town to buy it. I said I could not because my husband is not here. It was the doctor himself who took my prescription to go to the city to get the medicine and come back.”*

*(FGD, Mothers of children under 5)*

## 4. Discussion

To the best of our knowledge, this is the first study to provide evidence of the feasibility of field deployment of an MFT strategy at PHFs level. The study showed that the deployment was well received by both population and HWs, and resulted in a high compliance of HWs with the guidelines. The adherence of caregivers or patients to the dosing regimens was very high, as the drugs were perceived as highly efficacious.

Eighty-six percent of malaria patients received the appropriate ACT as per MFT guidelines, and 72.7% received the correct ACT at the correct dose; showing the HWs’ ability to comply with the MFT strategy. The good adherence of HWs to the MFT strategy guidelines may be explained by the simplicity and good documentation of the intervention, and the active involvement of key players in the health system (at central, intermediate, and peripheral levels) at all steps of the deployment. Indeed, the project was carefully embedded into the country health system using the same supply and distribution channel of ACTs. Furthermore, the subsidizing of new ACTs, their assured availability, considerable efforts for training, sensitization, supervision, and monitoring before and during the intervention, have all certainly enhanced the adherence of HWs and communities to the new strategy. We found that about 27% of confirmed cases were not adequately managed. This high proportion of non-adherence to the guidelines underlines the need for sustained monitoring of HWs practices, strengthening training and sensitization through periodic refresher training sessions, and improving the key actors’ commitment during the large-scale implementation of MFT.

Several MFT strategy options exist [24,25,27], and deciding on which one to deploy should be based on the specific health system organization. In our context in Burkina, the partitioning of ACT markets by segments was the most suitable option in the public sector. The other options previously described would have been very challenging given factors such as the low utilization rate of CHWs as the preferred source of care (<10%) [30] and the high uncertainty about private sector compliance. The desired uptake would have not been achieved [27]. Targeting segments of the population (children under 5 years of age, patients 5 years old and above, and pregnant women) is part of the health system routine practices, and hence was the most viable option [31]. Indeed, when introducing a national free healthcare policy for children under 5 and pregnant women, HWs adapted their practices accordingly [31]. This responsive attitude was seen during this project with no resistance to change reported. 

Our findings revealed that a large fraction of febrile patients was tested for malaria (79.1%) and nearly all (98.4%) of those tested positive received WHO-recommended treatment. Still, this testing rate is below the NMCP 100% target goal [32]. All PHFs were supplied with malaria RDTs. Thus, the lack of testing could potentially be related to HWs workload, lack of adherence to the guidelines or inadequate documentation of testing in the register. Very few cases (1.6%) of patients who tested positive were not treated. This finding is consistent with previous reports from Burkina Faso [33]. In addition, a few malaria RDT-negative patients (2.1%) received an ACT. A similar non-compliance of HWs to RDT negative results was reported in Uganda [34]. The use of RDT as malaria diagnosis test is known to be associated with non-adherence to treatment guidelines; owing potentially to the fact that RDTs may remain positive long time after a cured malaria episode [35].

Communities appreciated the strategy and adhered to it. The perceived efficacy of the two new ACTs (PYR-AS and DHA-PQP) associated with low frequencies of side effects, the availability of drugs, and the fact that the intervention was performed by the HWs whom they knew and trusted were identified as factors associated with this adherence. The acceptability of the MFT strategy was reflected by an increase in PHF use as the communities’ first source of care for febrile illness. In contrast, the proportion of patients seeking care within 24 h of fever onset decreased after the intervention, reflecting the need to support the program with sensitization and social behaviour change activities. Community members explained that they quickly recovered after taking ACTs prescribed by HWs. The perceived efficacy is a strong predictor of communities’ treatment adherence [36]. Although some people reported the occurrence of side effects following drugs intake, none were serious enough to discourage them in their positive opinion about the intervention. The distribution and availability of antimalarials for the three segments, as well as their relatively low cost (as subsidized), were highly appreciated by the populations. Many studies have previously reported that the removal of financial barriers to health services use increases communities’ attendance at PHF for care [31,37]. Regardless of the MFT option selected, subsidizing the cost of ACTs should be used to reduce affordability issues. 

The overall reported adherence to the treatment regimen was 82.1%. Similar adherence levels were previously reported in patients treated with ACTs [30]. The achieved adherence could be attributed to the effects of sensitization and the instructions (for 98.3% of patients) given by the HWs, and reflect the communities’ positive perception of the intervention. Our findings suggested that adherence was better for PYR-AS or DHA-PQP compared to AL. Higher adherence to DHA-PQP compared to AL was also reported in Malawi [38]. This appeared to be mainly influenced by the regimen of PYR-AS or DHA-PQP, which must be taken once daily whereas AL requires two intakes. In addition, the taste of drugs and the available paediatric formulation of PYR-AS in sachets seemed to have also positively affected the compliance. Some cases were not treated adequately, either by HWs or due to non-adherence of patient. Because both under-dosing and overdosing have negative consequences on malaria control and elimination [38,39], sensitization of communities and HWs on compliance to treatment regimen needs to be strengthened.

The drug stock management was satisfactory during the intervention. No drug stock- out was reported in the district store during the study period conversely to the HFs that experienced study drug stock-outs more frequently during the malaria peak period (September to November). The data suggest that the workload of GDSMs increases at malaria peak period, resulting in inadequacy of drug use and inventory. The stock-outs were confirmed by the communities expressing their experiences when attending PHFs. The low stock-out rates reported in this study refer to the context of Burkina Faso, which had a low stock-out rate before this pilot study implementation [40]. Our findings may not apply to malaria-endemic countries with a high stock-out rate. An efficient implementation of the MFT strategy requires continuous availability of all drugs used in the program. Frequent stock-outs of the drug dedicated to a specific segment of the population may affect compliance. 

### Strengths and Limitations

The study findings should be considered in the context of the following limitations and strengths. The mixed-method design provides some confidence on the robustness of the evidence generated. This could support a policy recommendation of the MFT approach as a cornerstone antimalarial resistance mitigation strategy.

The study has several limitations: Firstly, this pilot study was restricted to the PHFs only. Therefore, the findings may not be applicable if private health facilities and the community-based cases management are to be considered. Secondly, recall and social desirability bias may be associated with household surveys with the 4-week recall period and the self-reported data collected at the community level.

## 5. Conclusions

The MFT strategies have been suggested to mitigate the emerging partial artemisinin resistance in Africa. This was the first large-scale field project on the implementation of an MFT strategy for uncomplicated malaria treatment, and it has shown to be operationally feasible and highly acceptable by stakeholders in the public sector. This study provides evidence to support the simultaneous use of multiple first-line artemisinin combination therapies in malaria-endemic countries such as Burkina Faso. For a successful scale up of this strategy, the involvement of key stakeholders throughout will play a pivotal role. Sustained education and training, continuous availability of drugs are keys for success.

## Figures and Tables

**Table 1:** Sociodemographic characteristics of study participants during the HF-based survey.

	Segment I: Children < 5 Years	Segment II: 5 Years and above	Segment III: Pregnant Women	Total
	Frequency (%)	Frequency (%)	Frequency (%)	Frequency (%)
**Patients age group**	905 (45.1)	1012 (50.4)	91 (4.5)	2008 (100)
***Mean age (SD)***	*1.85 (1.3)*	*18.7 (15.7)*	*24.5 (6.1)*	*11.4 (14.2)*
**Gender**				
Male	459 (50.7)	464 (45.8)	0 (0.0)	923 (46.0)
Female	446 (49.3)	548 (54.2)	91 (100.0)	1085 (54.0)
**Residence area**				
Rural	568 (62.8)	591 (58.4)	42 (46.2)	1201 (59.8)
Urban	337 (37.2)	421 (41.6)	49 (53.8)	807 (40.2)
**Distance travelled for care**				
<5 km	620 (68.4)	727 (71.9)	68 (74.7)	1415 (70.5)
5–10 km	160 (17.7)	120 (11.9)	7 (7.7)	287 (14.3)
>10 km	126 (13.9)	164 (16.2%)	16 (17.6)	306 (15.2)

**Table 2:** HW compliance with MFT strategy guidelines and patient adherence to ACT regimen.

	Segment I: Children <5 Years	Segment II: Individuals ≥5 Years	Segment III: Pregnant Women	Total
	Frequency (%)	Frequency (%)	Frequency (%)	Frequency (%)
**HW performance in testing febrile patients for malaria**				
**Malaria RDT test performed**	***N = 906***	***N = 1011***	***N = 91***	***N = 2008***
Yes	706 (77.9)	813 (80.4)	69 (75.8)	1588 (79.1)
No	200 (22.1)	198 (19.6)	22 (24.2)	420 (20.9)
**Malaria RDT result**	***N = 706***	***N = 813***	***N = 69***	***N = 1588***
Positive	408 (57.8)	589 (72.5)	43 (62.3)	1040 (65.5)
Negative	292 (41.4)	211 (25.9)	26 (37.7)	529 (33.3)
Missing information	6 (0.8)	13 (1.6)	0 (0.0)	19 (1.2)
**Treatment of patients**				
RDT positive *	***N* = 401**	***N* = 578**	***N* = 41**	***N* = 1020**
PYR-AS	347 (86.5)	28 (4.8)	0 (0.0)	375 (36.8)
DHA-PQP	9 (2.3)	464 (80.3)	0 (0.0)	473 (46.4)
AL	36 (9.0)	81 (14.0)	21 (51.2)	138 (13.5)
QUININE	0 (0.0)	0 (0.0)	18 (43.9)	18 (1.8)
No treatment	9 (2.2)	5 (0.9)	2 (9.9)	16 (1.6)
**RDT negative**	***N = 292***	***N = 211***	***N = 26***	***N = 529***
Treated with ACT	4 (1.4)	4 (2.0)	2 (7.7)	110 (1.9)
No treatment	288 (98.6)	206 (97.6)	24 (92.3)	518 (98.1)
**RDT not done**	***N = 200***	***N = 198***	***N = 22***	***N = 420***
Treated	52 (26.0)	95 (48.0)	6 (27.3)	153 (36.4)
No treatment	148 (74.0)	103 (52.0)	16 (72.7)	267 (63.6)
**HWs compliance with MFT strategy**				
Appropriate ACT given **	348 (86.8)	493 (85.3)	37 (90.2)	878 (86.1)
Correctly treated ***	275 (74.1)	377 (70.5)	36 (87.8)	688 (72.7)
**Adherence to ACT regimen from the household survey**	***N = 404***	***N = 707***	***N = 24***	***N = 1135***
Adhere to 3-days regimen for any ACT	335 (82.9)	577 (81.6)	20 (83.3)	932 (82.1)
Adhere to 3-days regimen for PYR-AS	296 (85.3)	16 (84.2)	0 (0.0)	312 (85.3)
Adhere to 3-days regimen for DHA-PQP	12 (92.3)	364 (84.9)	0 (0.0)	376 (85.1)
Adhere to 3-days regimen for AL	27 (61.4)	197 (76.1)	20 (83.3)	244 (74.6)

* We excluded positive RDT associated with severe malaria (n = 20). ** Appropriate treatment refers to the confirmed uncomplicated malaria treated with predefined and allocated ACT according to the MFT strategy. One child under 5 weighing more than 20 kg was treated with DHA-PQP and 29 patients aged 5 years or more, weighing less than 17 kg, were treated either with PYR-AS or AL; these were considered as good compliance. *** Correct treatment is defined by patients having received appropriate treatment with the correct dose.

**Table 3:** Sociodemographic characteristics of study participants during the household surveys.

	Before Intervention				After Intervention			
	Children < 5 Years	≥5 Years	Pregnant Women	Total	Children < 5 Years	≥5 Years	Pregnant Women	Total
***Total of participants interviewed***	***462 (33.1)***	***898 (64.4)***	***34 (2.4)***	***1394 (100)***	***485 (33.5)***	***933 (64.4)***	***30 (2.1)***	***1448 (100)***
***Gender***								
Male, n (%)	240 (51.9)	350 (39.0)	0 (0.0)	590 (42.3)	252 (52.0)	406 (43.5)	0 (0.0)	658 (45.4)
Female, n (%)	222 (48.1)	548 (61.0)	34 (100.0)	804 (57.7)	232 (48.8)	514 (55.1)	30 (100.0)	776 (53.6)
Missing data	0 (0.0)	0 (0.0)	0 (0.0)	0 (0.0)	1 (0.2)	13 (1.4)	0 (0.0)	14 (1.0)
**Education level of respondents**								
None	370 (80.1)	570 (63.5)	22 (64.7)	962 (69.0)	430 (88.7)	527 (56.5)	20 (66.7)	977 (67.5)
Informal education	35 (7.6)	72 (8.0)	4 (11.8)	111 (8.0)	9 (1.9)	36 (3.9)	2 (6.7)	47 (3.3)
Primary school	27 (5.8)	150 (16.7)	4 (11.8)	181 (13.0)	15 (3.1)	224 (24.0)	4 (13.3)	243 (16.8)
Secondary and higher	18 (3.9)	98 (10.9)	4 (11.8)	120 (8.6)	17 (3.5)	132 (14.1)	4 (13.3)	153 (10.6)
Missing data	12 (2.6)	8 (0.9)	0 (0.0)	20 (1.4)	14 (2.9)	14 (1.5)	0 (0.0)	28 (1.9)
**Occupation of respondents**								
Farmer	374 (81.0)	666 (74.2)	23 (67.7)	1063 (76.3)	368 (75.9)	608 (65.2)	18 (60.0)	994 (68.6)
Housewife	50 (10.8)	47 (5.2)	5 (14.7)	102 (7.3)	93 (19.2)	235 (25.2)	11 (36.7)	339 (23.4)
Employee/Merchant	25 (5.4)	71 (7.9)	3 (8.8)	99 (7.1)	12 (2.5)	40 (4.3)	0 (0.0)	52 (3.6)
Student	4 (0.9)	54 (6.0)	2 (5.9)	60 (4.3)	2 (0.4)	17 (1.8)	1 (3.3)	20 (1.4)
Others	7(1.5)	27 (3.0)	0 (0.0)	34 (2.4)	7 (1.4)	26 (2.8)	0 (0.0)	33 (2.3)
Missing data	2 (0.4)	33 (3.7)	1 (2.9)	36 (2.6)	3 (0.6)	7 (0.8)	0 (0.0)	10 (0.7)
**Household size**								
≤6	109 (23.6)	216 (24.1)	16 (47.1)	341 (24.5)	156 (32.2)	237 (25.4)	8 (26.7)	401 (27.7)
>6	353 (76.4)	682 (75.9)	18 (52.9)	1053 (75.5)	329 (67.8)	696 (74.6)	22 (73.3)	1047 (72.3)

**Table 4:** Effect of MFT strategy on community care-seeking behaviours and malaria case management.

	Before Intervention				After Intervention			
	Children < 5 Years	≥5 Years	Pregnant Women	Total	Children < 5 Years	≥5 Years	Pregnant Women	Total
***Total of participants interviewed***	***432 (33.5)***	***898 (64.4)***	***34 (2.4)***	***1394 (100)***	***485 (33.5)***	***933 (64.4)***	***30 (2.1)***	***1448 (100)***
***Care-seeking behaviours***								
Sought care, n (%)	454 (98.3)	880 (98.0)	32 (94.1)	1366 (98.0)	*480 (99.0)*	*913 (97.9)*	*30 (100)*	*1423 (98.3)*
Care-seeking within 24 h, n (%)	311 (68.5)	573 (65.1)	20 (62.5)	**904 (66.5)**	256 (53.3)	487 (53.3)	10 (33.3)	**753 (54.3)**
***Sources of providers for care***								
Public health facilities, n (%)	384 (84.6)	570 (64.8)	28 (87.5)	**982 (72.0)**	407 (84.8)	693 (76.0)	29 (96.7)	**1129 (79.4)**
Private HF/NGO, n (%)	3 (0.6)	57 (6.5)	1 (3.1)	**61 (4.5)**	9 (1.9)	18 (2.0)	0 (0.0)	**27 (1.9)**
Community health workers, n (%)	25 (5.5)	102 (11.6)	2 (6.3)	**129 (9.5)**	32 (6.7)	63 (6.9)	0 (0.0)	**95 (6.7)**
Family stock, n (%)	26 (5.7)	53 (6.0)	0 (0.0)	79 (5.8)	20 (4.2)	59 (6.5)	0 (0.0)	79 (5.6)
Traditional healer, n (%)	7 (1.4)	11 (2.4)	1 (3.1)	32 (2.3)	7 (1.5)	37 (4.1)	0 (0.0)	44 (3.1)
Private pharmacy, n (%)	2 (0.4)	9 (2.0)	0 (0.0)	40 (2.9)	2 (0.4)	24 (2.6)	1 (3.3)	27 (1.9)
Street vendor, n (%)	5 (1.1)	13 (2.8)	0 (0.0)	**35 (2.6)**	3 (0.6)	14 (1.5)	0 (0.0)	**17 (1.2)**
***Malaria RDT test performed and treatment practices***								
Tested for malaria, n (%)	348 (75.3)	619 (68.9)	25 (73.5)	**992** **(71.2)**	424 (87.6)	720 (77.6)	27 (93.1)	**1171** **(81.3)**
Positive malaria test, n (%)	326 (94.2)	610 (98.7)	22 (88.0)	958 (96.9)	408 (99.0)	707 (99.2)	25 (92.6)	1140 (98.9)
Treated with ACT from any providers, n (%)	319 (71.9)	659 (77.3)	15 (48.4)	**993 (74.8)**	414 (85.4)	726 (77.8)	24 (80.0)	**1164 (80.4)**
Malaria treatment advice given to patient, n (%)	311 (98.4)	640 (98.3)	15 (100.0)	966 (98.4)	400 (97.6)	699 (98.6)	24 (100.0)	1123 (98.3)
Adherence to 3-day ACT treatment regimen, n (%)	244 (77.2)	295 (87.8)	12 (80.0)	830 (84.3)	335 (82.9)	577 (81.6)	20 (83.3)	932 (82.1)

In bold, statistically significant outcomes with *p* < 0.05 comparing pre- and post-intervention.

**Table 5:** Occurrence of adverse events after treatment with study drug.

	PYR-AS (n = 308)	DHA-PQP (n = 374)	AL (n = 267)	Total (n = 949)
**Any adverse event**	**25 (8.1)**	**35 (9.4)**	**39 (14.6)**	99 (10.4)
Vomiting	11 (3.6)	9 (2.4)	17 (6.4)	37 (3.9)
Pruritus	3 (1.0)	5 (1.3)	5 (1.9)	13 (1.4)
Anorexia	1 (0.3)	7 (1.9)	3 (1.2)	11 (1.2)
Diarrhoea	3 (1.0)	3 (0.8)	3 (1.1)	9 (1.0)
Somnolence	3 (1.0)	3 (0.8)	3 (1.1)	9 (1.0)
Abdominal pain	1 (0.3)	3 (0.8)	1 (0.4)	5 (0.5)
Headache	0 (0.0)	0 (0.0)	4 (1.5)	4 (0.4)
Other	3 (1.0)	5 (1.3)	3 (1.1)	11 (1.2)

## Data Availability

Data are available on reasonable request.

## Supplementary Materials

The following supporting information can be downloaded at: https://www.mdpi.com/article/10.3390/tropicalmed8040195/s1, Table S1: Study drugs regimen used in this MFT pilot programme; Table S2: Sociodemographic characteristics of study participants during the qualitative survey.
