# Supplementary material for: Feasibility and Acceptability of a Strategy Deploying Multiple First-Line Artemisinin-Based Combination Therapies for Uncomplicated Malaria in the Health District of Kaya, Burkina Faso
Source: Trop Med Infect Dis. 2023 Mar 28;8(4):195. doi: 10.3390/tropicalmed8040195 (PMC10145444; doi:10.3390/tropicalmed8040195)
Supplement: Supplementary file 1 [file tropicalmed-08-00195-s001.zip › Supplementary Table S1.pdf]

<u>Supplementary Table S1</u>: Study drugs regimen used in this MFT pilot programme

Pyronaridine-Artesunate

Recommended dosage of fixed combination of Pyronaridine-Artesunate granule 20/60 mg for children under five years of age

Weight	Presentations	Day 1	Day 2	Day 3
**5-<8 kg**	Pyronaridine 20 mg<br>Artesunate 60 mg	1 sachet	1 sachet	1 sachet
**8-<15kg**	Pyronaridine 20 mg<br>Artesunate 60 mg	2 sachets	2 sachets	2 sachets
**15-<20kg**	Pyronaridine 20 mg<br>Artesunate 60 mg	3 sachets	3 sachets	3 sachets

Dihydroartemisinin-Piperaquine

Recommended dosage of fixed combination Dihydroartemisinine-Piperaquine (40/320) for five years of age children and above

Weight (age)	Presentation/dosage	Day 1	Day 2	Day 3
17 -<25 kg (5-8 years)	Dihydroartemisinin 40mg+<br>Piperaquine 320mg	1+1/2 tablets	1+1/2 tablets	1+1/2 tablets
25 -<36 kg (8 -15 years)	Dihydroartemisinin 40mg+<br>Piperaquine 320mg	2 tablets	2 tablets	2 tablets
36 -<60 kg (≥15 years)	Dihydroartemisinin 40mg+<br>Piperaquine 320mg	3 tablets	3 tablets	3 tablets
60 -<80 kg (≥15 years)	Dihydroartemisinin 40mg+<br>Piperaquine 320mg	4 tablets	4 tablets	4 tablets
≥80kg (≥15 years)	Dihydroartemisinin 40mg+<br>Piperaquine 320mg	5 tablets	5 tablets	5 tablets

Artemether-lumefantrine

Recommended dosage of fixed combination Artemether-Lumefantrine (80/480 mg) for pregnant women

Weight (age)	Presentations	Day 1	Day 2	Day 3
≥35 kg<br>(≥ 12 years)	Artemether 80 mg<br>Lumefantrine 480 mg	1tb x 2/D	1tb x 2/D	1tb x 2/D
Adult	Artemether 80 mg<br>Lumefantrine 480 mg	1tb x 2/D	1tb x 2/D	1tb x 2/D

D : day tb : tablet
