# Supplementary material for: Feasibility and Acceptability of a Strategy Deploying Multiple First-Line Artemisinin-Based Combination Therapies for Uncomplicated Malaria in the Health District of Kaya, Burkina Faso
Source: Trop Med Infect Dis. 2023 Mar 28;8(4):195. doi: 10.3390/tropicalmed8040195 (PMC10145444; doi:10.3390/tropicalmed8040195)
Supplement: Supplementary file 1 [file tropicalmed-08-00195-s001.zip › Supplementary Table S2.pdf]

***Supplementary Table S2*: Sociodemographic characteristics of study participants during the qualitative survey**

	Number	Percentage (%)
**Age of respondents (years)**		
≤ 25	73	19.0
26-35	104	27.1
36-55	171	44.5
> 55	36	9.4
**Sex of respondents**		
Female	163	42.5
Male	221	57.5
**Residence area of respondents**		
Rural	316	82.3
Urban	68	17.7
**Education level**		
None	197	51.3
Informal education	24	6.2
Primary	31	8.1
Secondary and higher	132	34.4
